# Development and validation of a prognostic nomogram among patients with acute exacerbation of chronic obstructive pulmonary disease in intensive care unit

**DOI:** 10.1186/s12890-022-02100-0

**Published:** 2022-08-09

**Authors:** Jiang-Chen Peng, Wen-Wen Gong, Yan Wu, Tian-Yi Yan, Xiao-Yan Jiang

**Affiliations:** 1 Department of Critical Care, Ren Ji Hospital, School of Medicine, Shanghai Jiao Tong University, 160 Pujian Road, Shanghai, 200127 China; 2 Department of Critical Care, Shanghai Baoshan Luodian Hospital, 121 Luoxi Road, Baoshan District, Shanghai, 201908 China; 3 Department of Emergency, Shanghai Baoshan Luodian Hospital, 121 Luoxi Road, Baoshan District, Shanghai, 201908 China; 4 Department of Nursing, Shanghai Baoshan Luodian Hospital, 121 Luoxi Road, Baoshan District, Shanghai, 201908 China; 5 Department of Nursing, Shanghai Baoshan Luodian Hospital, 121 Luoxi Road, Shanghai, 201908 China

**Keywords:** AECOPD, Nomogram, Predictive model, 30-day mortality, Intensive care unit

## Abstract

**Background:**

Acute exacerbation of Chronic Obstructive Pulmonary Disease (AECOPD) contributes significantly to mortality among patients with COPD in Intensive care unit (ICU). This study aimed to develop a nomogram to predict 30-day mortality among AECOPD patients in ICU.

**Methods:**

In this retrospective cohort study, we extracted AECOPD patients from Medical Information Mart for Intensive Care III (MIMIC-III) database. Multivariate logistic regression based on Akaike information criterion (AIC) was used to establish the nomogram. Internal validation was performed by a bootstrap resampling approach with 1000 replications. The discrimination and calibration of the nomogram were evaluated by Harrell’s concordance index (C-index) and Hosmer–Lemeshow (HL) goodness-of-fit test. Decision curve analysis (DCA) was performed to evaluate its clinical application.

**Results:**

A total of 494 patients were finally included in the study with a mean age of 70.8 years old. 417 (84.4%) patients were in the survivor group and 77 (15.6%) patients were in the non-survivor group. Multivariate logistic regression analysis based on AIC included age, pO_2_, neutrophil-to-lymphocyte ratio (NLR), prognostic nutritional index (PNI), invasive mechanical ventilation and vasopressor use to construct the nomogram. The adjusted C-index was 0.745 (0.712, 0.778) with good calibration (HL test, *P* = 0.147). The Kaplan–Meier survival curves revealed a significantly lower survival probability in the high-risk group than that in the low-risk group (*P* < 0.001). DCA showed that nomogram was clinically useful.

**Conclusion:**

The nomogram developed in this study could help clinicians to stratify AECOPD patients and provide appropriate care in clinical setting.

## Introduction

Chronic Obstructive Pulmonary Disease (COPD) is a common chronic respiratory disease featured by persistent respiratory symptoms and airflow limitation [1]. COPD is an important public health challenge and associated with high mortality worldwide. According to the WHO, it is estimated that global COPD will rise to the third leading cause of death in 2030, with the corresponding economic burden ranking the fifth [2]. Acute exacerbation of COPD (AECOPD) is defined as an acute worsening of respiratory symptoms in COPD which require additional therapy [1]. AECOPD contributes significantly to mortality among patients with COPD [3], especially for those who require intensive care unit (ICU) admission with a high mortality rate of 16.9% to 48.8% [4, 5]. The severity of exacerbations reflected by clinical results is strongly correlated with patient prognosis. Whereas accurate decision-making and prompt treatment would predict a better prognosis, it is important to identify the factors that is able to predict outcomes in AECOPD patients. Although several studies have investigated independent factors to predict mortality due to AECOPD [6–9], few study especially focused on patients with AECOPD in ICU.

Medical Information Mart for Intensive Care III (MIMIC III) database can provide a wealth of clinical data to be routinely analyzed. The purpose of our study was to determine independent factors affecting mortality for patients with AECOPD in MIMIC III database by nomogram. Nomograms, user-friendly instrument with visualized prediction outcomes, are popular prognostic tools with the ability to predict clinical events by integrating potential risk factors [10]. Nomogram could be easily applied in clinical practice to identify high-risk patients and guide decision-making. Hence, we intend to develop and internally validate a nomogram to predict 30-day mortality after admission to ICU among AECOPD patients.

## Material and methods

### Data source

We extracted the data of this retrospective study from MIMIC-III version 1.4 (MIMIC-III v1.4) database. MIMIC-III is a large, open, and public database, containing more than 50,000 patients admitted to the ICU at Beth Israel Deaconess Medical Center from 2001 to 2012 [11]. We accessed the MIMIC-III after completion of the Protecting Human Research Participants exam. The establishment and employment of this database were approved by the Institutional Review Boards of the Massachusetts Institute of Technology and Beth Israel Deaconess Medical Center. No informed consent was required since all the data were de-identified.

### Study population, data extraction and outcome

Adult patients (≥ 18 years old) with the diagnosis of AECOPD were selected from the database. The definition of AECOPD was based on the International Classification of Diseases, 9th edition (ICD-9) code 491.21. For patients with multiple hospitalizations, only the first hospitalization was enrolled. Other exclusion criteria included length of ICU stay < 48 h and missing data > 10%.

Data were extracted from MIMIC-III database through Structured Query Language [12]. The data upon admission to ICU were recorded, including age, gender, full blood count (white blood cell (WBC) count, neutrophil count, lymphocyte count, platelet count, hemoglobin), laboratory values (serum albumin, alanine transaminase (ALT), aspartate transaminase (AST), serum creatinine (sCr), serum blood urea nitrogen (BUN), serum sodium, serum potassium, serum calcium), arterial blood gas (pH, partial pressure of oxygen (pO_2_), partial pressure of carbon dioxide (pCO_2_), bicarbonate), vital signs (temperature, mean atrial pressure (MAP), heart rate, respiratory rate), comorbidities (hypertension, diabetes mellitus (DM), coronary heart disease (CHD), chronic renal disease (CKD), maligancy), treatment therapy (invasive mechanical ventilation (IMV) and vasopressor) and clinical severity scales (Sequential Organ Failure Assessment (SOFA) score and Simplified Acute Physiology Score II (SAPS II)). The neutrophil-to-lymphocyte ratio (NLR) was defined as the absolute count of neutrophils divided by the absolute count of lymphocytes. The platelet-to-lymphocyte ratio (PLR) was defined as the absolute count of platelets divided by the absolute count of lymphocytes. The prognostic nutritional index (PNI) value was calculated as the following equation: 10 × serum albumin (g/dL) + 0.005 × total lymphocyte count (mm^3^). For missing variables, predictive mean matching was used to impute numeric features. The primary outcome was 30-day all-cause mortality after admission to ICU.

### Statistical analysis

Continuous variables are presented as the mean ± standard deviation (SD) for normal distribution and as the median and interquartile range (IQR) for skewed distribution. Normal distributions were confirmed by Agostino tests. Continuous variables were compared by unpaired Student's test or Mann–Whitney *U*-test. Categorical variables were compared using the χ^2^-test or Fisher exact test as appropriate. The median difference (MD) were analyzed by Hodges-Lehmann estimate along with 95% confidence interval (CI).

Univariate Cox proportional hazard analysis was performed to explore the potential confounders associated with 30-day mortality. Subsequently, variables with *P* values < 0.1 in univariate were used to establish multivariate Cox regression by the backward step-down process based on the Akaike information criterion (AIC) to estimate the hazard ratio (HR) and 95% CI. The final model minimized the score of AIC in order to have fewest variables. The variance inflation factor (VIF) was calculated to detect the potential collinearity between continuous variables. When VIF > 10, collinearity was considered to exist and it will be solved by regularization. Nomogram was constructed based on the multivariate Cox regression results to visualize the model [13]. The Harrell’s concordance index (C-index) and receiver operating characteristic (ROC) curve were used to evaluate the discrimination ability of the nomogram. The nomogram was then calibrated graphically by visual examination of the calibration plot with the Hosmer–Lemeshow (HL) goodness-of-fit test. Internal validation of the final multivariate model was performed using a bootstrap resampling approach with 1000 replications. Decision curve analysis (DCA) was performed to assess the clinical usefulness of the nomogram by quantifying the standardized net benefits at different threshold probabilities [14]. Finally, according to the median of risk score, all patients were divided into the high-risk and low-risk groups, and the survival curve with a log-rank test was used to verify the prognostic value of nomogram.

All analyses were conducted using R software (version 3.6.3) and two-sided *p* values less than 0.05 were considered statistically significant in each statistical analysis.

## Results

### Baseline characteristics of the included patients

**Fig. 1:**
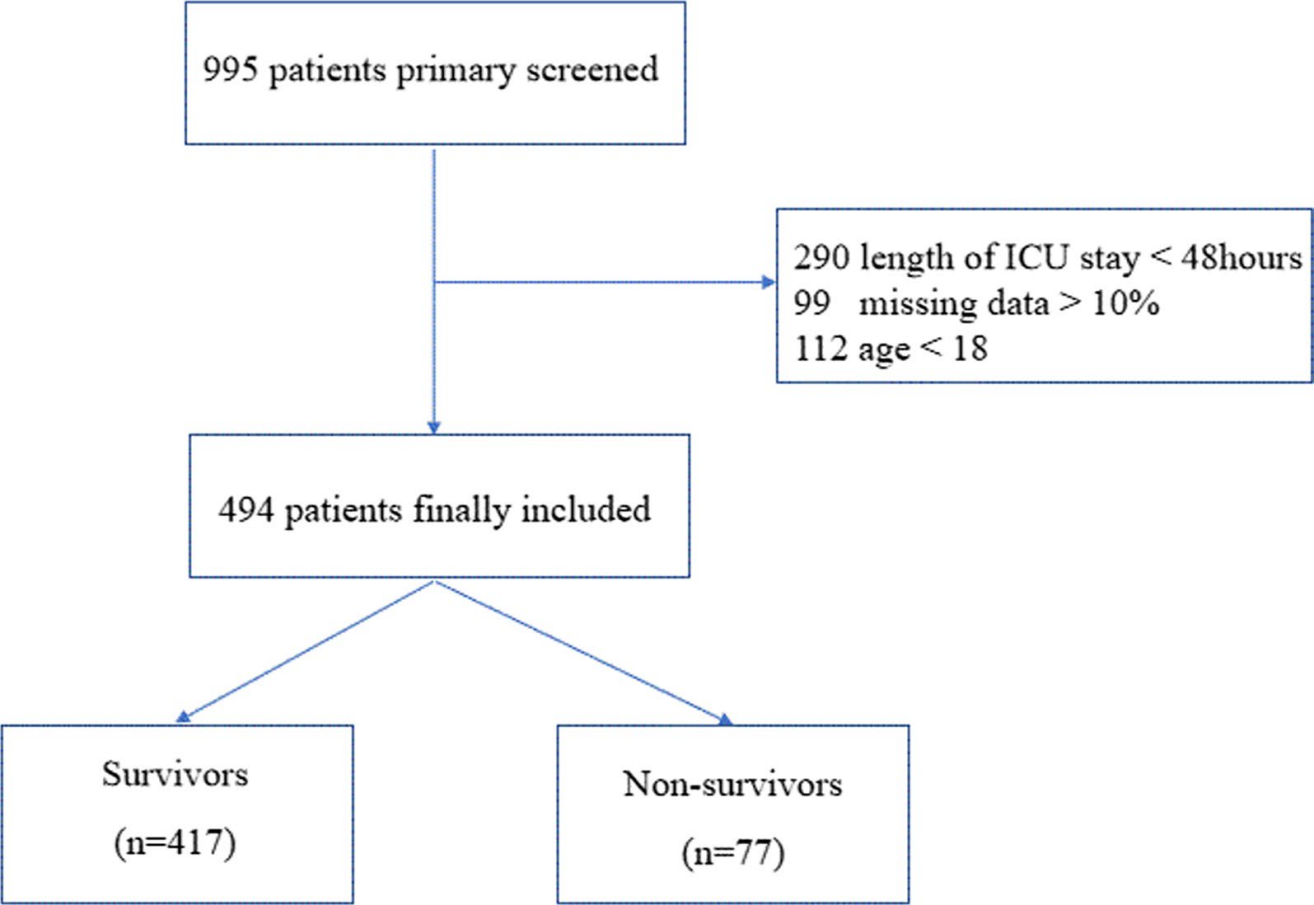
The flow chart of the included population

995 patients with AECOPD in the MIMIC-III database were primary screened. 501 patients were excluded due to length of ICU stay < 48 h, missing data > 10% or age < 18 (Fig. 1). Finally, a total of 494 patients with AECOPD were included in our study, with a mean age of 70.8 years old and 50.2% male. The baseline characteristics of the study population were shown in Table 1. 417 (84.4%) patients were in the survivor group and 77 (15.6%) patients were in the non-survivor group. Non-survivors tended to be elder compared with the survivors (75.3 ± 8.6 years old vs. 69.9 ± 10.5 years old, *P* < 0.001). Deceased patients had significantly higher levels of neutrophil count (MD = 1600/μL, 95%CI, 300 to 2900/μL), BUN (MD = 4.0 mmol/L, 95%CI, 1.0 to 8.0 mmol/L), NLR (MD = 3.8, 95%CI, 1.5 to 7.1) and PLR (MD = 63.4, 95%CI, 9.3 to 119.9). While, the levels of lymphocyte count (MD = − 2600/μL, 95%CI, − 4000 to − 1000/μL), serum albumin (MD = − 0.3 g/dL, 95%CI, − 0.5 to − 0.1 g/dL), partial pressure of oxygen (MD = -16 mmHg, 95%CI, − 30 to − 2 mmHg) and PNI (MD = − 3.2, 95%CI, − 4.9 to − 1.6) were significantly lower in non-survivors. Clinical severities, such as SAPS II (MD = 6, 95%CI, 3 to 9) and SOFA (MD = 1, 95%CI, 0 to 2), tended to be more severe in the non-survivor group when compared with the survivor group. Non-survivors were more likely to be treated with IMV (61% vs. 46.3%, *P* = 0.002) and vasopressor (54.5% vs. 32.4%, *P* < 0.001). In terms of vital signs, there were no significant differences between the two groups.

**Table 1:** Baseline and clinical characteristics of the study population

Characteristics	All (n = 494)	Survivors (n = 417)	Non-survivors (n = 77)	*P* value
Age, years (mean ± SD)	70.8 ± 10.4	69.9 ± 10.5	75.3 ± 8.6	< 0.001^a^
*Gender, n (%)*				
Male	248 (50.2)	210 (50.4)	38 (49.4)	0.871^b^
Female	246 (49.8)	207 (49.6)	39 (50.6)	0.871^b^
*Laboratory test, median (IQR)*				
WBC count (10^3^/μL)	11.1 (8.0, 15.6)	10.9 (8.0, 15.1)	12.0 (9.1, 17.7)	0.084^c^
Neutrophil count (10^3^/μL)	9.2 (6.2, 13.2)	8.8 (6.1, 13.0)	10.4 (7.2, 15.1)	0.019^c^
Lymphocyte count (10^3^/μL)	0.9 (0.5, 1.6)	0.9 (0.5, 1.6)	0.8 (0.4, 1.2)	0.015^c^
Platelet count (10^3^/μL)	236 (175, 308)	239 (175, 307)	224 (162, 309)	0.434^c^
Hemoglobin (g/dL)	10.8 (9.6, 12.3)	10.9 (9.6, 12.4)	10.6 (9.5, 11.5)	0.145^c^
Serum albumin (g/dL)	3.2 (2.7, 3.6)	3.3 (2.8, 3.7)	2.9 (2.6, 3.4)	< 0.001^c^
Serum sodium (mmol/L)	138 (134, 141)	137 (134, 141)	137 (134, 140)	0.592^c^
Serum potassium (mmol/L)	4.2 (3.7, 4.7)	4.2 (3.7, 4.7)	4.1 (3.5, 4.6)	0.080^c^
Serum calcium (mmol/L)	1.13 (1.07, 1.19)	1.13 (1.10, 1.19)	1.13 (1.10, 1.16)	0.982^c^
pH	7.34 (7.25, 7.40)	7.34 (7.26, 7.40)	7.32 (7.25, 7.40)	0.503^c^
pO_2_ (mmHg)	103 (74, 187)	107 (76, 192)	88 (61, 167)	0.018^c^
pCO_2_ (mmHg)	67 (53, 72)	53 (44, 72)	52 (46, 71)	0.810^c^
Bicarbonate (mmol/L)	28 (25, 33)	28 (25, 33)	30 (25,34)	0.574^c^
ALT (IU/L)	23 (15, 40)	23 (15, 40)	21 (15, 40)	0.975^c^
AST (IU/L)	27 (18, 42)	26 (18, 42)	27 (17, 46)	0.968^c^
Serum creatinine (mg/dL)	1.0 (0.7, 1.4)	1.0 (0.7, 1.4)	1.0 (0.7, 1.3)	0.787^c^
BUN (mmol/L)	25 (18, 38)	24 (18, 36)	28 (21, 44)	0.021^c^
*Mean vital signs, median (IQR)*				
Temperature (℃)	36.7 (36.4, 37.1)	36.7 (36.3, 37.1)	36.7 (36.4, 37.1)	0.738^c^
MAP (mmHg)	77 (71, 85)	77 (71, 84)	75 (70, 83)	0.176^c^
Heart rate (min^−1^)	90 (80, 100)	89 (80, 99)	90 (81, 103)	0.142^c^
Respiratory rate (min^−1^)	20 (17, 23)	20 (17, 23)	20 (17, 23)	0.587^c^
*Comorbidities, n (%)*				
Hypertension	246 (49.8)	216 (51.8)	30 (39.0)	0.038^b^
DM	137 (27.7)	124 (29.7)	13 (16.9)	0.021^b^
CHD	112 (22.7)	94 (22.5)	18 (23.4)	0.352^b^
CKD	83 (16.8)	75 (18.0)	8 (10.4)	0.101^b^
Maligancy	131 (26.5)	108 (25.9)	23 (29.9)	0.468^b^
*Inflammatory indicators, median (IQR)*				
NLR	8.8 (5.0, 16.6)	8.8 (5.0, 16.6)	14.6 (6.6, 31.5)	< 0.001^c^
PLR	246.3 (133.9, 467.7)	237.5 (129.5, 436.9)	295.9 (175.6,593.2)	0.022^c^
PNI	33.3 (28.6, 37.6)	33.8 (29.2, 38.0)	30.1 (26.2, 35.4)	< 0.001^c^
*Scoring system, median (IQR)*				
SAPSII	39 (32, 47)	38 (31, 45)	44 (38, 51)	< 0.001^c^
SOFA	4 (2, 6)	4 (2, 6)	5 (4, 7)	0.007^c^
*Treatment, n (%)*				
IMV	240 (48.6)	193 (46.3)	47 (61.0)	0.002^b^
Vasopressor	177 (35.8)	135 (32.4)	42 (54.5)	< 0.001^b^

Normally distributed data are presented as the mean ± SD, non-normally distributed data are presented as median (IQR) and categorical variables are presented as n (%)

^a^The analysis was performed by using independent samples Student's T test

^b^The analysis was performed by using χ^2^-test

^c^The analysis was performed by using Mann–Whitney *U*-test

*ALT* alanine transaminase, *AST* aspartate transaminase, *BUN* blood urea nitrogen, *CHD* coronary heart disease, *CKD* chronic kidney disease, *DM* diabetes mellitus, *IMV* invasive mechanical ventilation, *MAP* mean atrial pressure, *NLR* neutrophil-to-lymphocyte ratio, *PLR* platelet-to-lymphocyte ratio, *PNI* prognostic nutritional index, *SAPSII* simplified acute physiology score II, *SOFA* sequential organ failure assessment, *WBC* white blood cell

### Univariate and multivariate prognostic analyses

Univariate Cox regression analysis showed that age (HR = 1.057, 95% CI, 1.030 to 1.085; *P* < 0.001), neutrophil count (HR = 1.034, 95% CI, 1.004 to 1.065; *P* = 0.026), serum albumin (HR = 0.547, 95% CI, 0.379 to 0.787; *P* = 0.001), NLR (HR = 1.009, 95% CI, 1.003 to 1.016; *P* = 0.006), PNI (HR = 0.941, 95% CI, 0.908 to 0.975; *P* < 0.001), SAPSII (HR = 1.037, 95% CI, 1.004 to 1.065; *P* = 0.026), SOFA (HR = 1.099, 95% CI, 1.020 to 1.184; *P* = 0.013), IMV (HR = 2.602, 95% CI, 1.339 to 5.057; *P* = 0.005) and vasopressor use (HR = 2.206, 95% CI, 1.408 to 3.455; *P* < 0.001) were significantly associated with 30-day mortality. After considering collinearity, multivariate Cox regression analysis based on AIC identified that age (HR = 1.066, 95% CI, 1.037 to 1.095; *P* < 0.001), pO_2_ (HR = 0.997, 95% CI, 0.995 to 0.999; *P* = 0.009), NLR (HR = 1.006, 95% CI, 1.001 to 1.013; *P* = 0.048), PNI (HR = 0.958, 95% CI, 0.923 to 0.994; *P* = 0.024), IMV (HR = 2.516, 95% CI, 1.265 to 5.005; *P* = 0.008) and vasopressor use (HR = 2.042, 95% CI, 1.267 to 3.292; *P* = 0.003) were independent risk factors for 30-day mortality (Table 2).

**Table 2:** Univariate and multivariate analysis of Cox-proportional hazards model for the risk of 30-day mortality

	Univariate (HR, 95% CI)	*P* value	Multivariate (HR, 95% CI)	VIF	*P* value
Age	1.057 (1.030, 1.085)	< 0.001	1.066 (1.037, 1.095)	1.3	< 0.001
Male	0.973 (0.622, 1.521)	0.904	**/**	–	–
*Laboratory tests*					
WBC count	1.025 (0.999, 1.052)	0.063	**/**	–	–
Neutrophil count	1.034 (1.004, 1.065)	0.026	**/**	–	**/**
Lymphocyte count	0.886 (0.709, 1.107)	0.287	**/**	–	–
Platelet count	1.000 (0.998, 1.002)	0.779	–	–	–
Hemoglobin	0.930 (0.829, 1.043)	0.213	–	–	–
Serum albumin	0.547 (0.379, 0.787)	0.001	–	–	–
Serum sodium	0.995 (0.954, 1.037)	0.799	–	–	–
Serum potassium	0.772 (0.580, 1.027)	0.076	–	–	–
Serum calcium	1.257 (0.056, 28.164)	0.885	–	–	–
pH	0.474 (0.059, 3.820)	0.483	–	–	–
pO_2_	0.998 (0.996, 1.000)	0.079	0.997 (0.995, 0.999)	1.1	0.009
pCO_2_	1.000 (0.990, 1.011)	0.957	–	–	–
Bicarbonate	1.010 (0.975, 1.045)	0.588	–	–	–
ALT	0.999 (0.997, 1.001)	0.409	–	–	–
AST	0.999 (0.997, 1.001)	0.484	–	–	–
Serum creatinine	1.058 (0.881, 1.270)	0.547	–	–	–
BUN	1.006 (0.997, 1.015)	0.192	–	–	–
*Mean vital signs*					
Temperature	0.934 (0.641, 1.361)	0.723	–	–	–
MAP	0.984 (0.962, 1.007)	0.175	–	–	–
Heart rate	1.012 (0.997, 1.028)	0.122	–	–	–
Respiratory rate	1.018 (0.964, 1.075)	0.524	–	–	–
*Comorbidities*					
Hypertension	0.635 (0.402, 1.004)	0.052	–	–	–
DM	0.710 (0.581, 1.025)	0.056	–	–	–
CHD	1.383 (0.697, 1.745)	0.225	–	–	–
CKD	0.564 (0.271, 1.173)	0.125	–	–	–
Malignancy	1.190 (0.731, 1.939)	0.484	–	–	–
*Inflammatory indicators*					
NLR	1.009 (1.003, 1.016)	0.006	1.006 (1.001, 1.013)	1.4	0.048
PLR	1.000 (1.000, 1.001)	0.108	–	–	–
PNI	0.941 (0.908, 0.975)	< 0.001	0.958 (0.923, 0.994)	1.0	0.024
*Scoring system*					
SAPSII	1.037 (1.020, 1.054)	< 0.001	–	–	–
SOFA	1.099 (1.020, 1.184)	0.013	–	–	–
*Treatment*					
MV	2.602 (1.339, 5.057)	0.005	2.516 (1.265, 5.005)	1.2	0.008
Vasopressor	2.206 (1.408, 3.455)	< 0.001	2.042 (1.267, 3.292)	1.4	0.003

*ALT* alanine transaminase, *AST* aspartate transaminase, *BUN* blood urea nitrogen, *CHD* coronary heart disease, *CKD,* chronic kidney disease, *DM* diabetes mellitus, *IMV* invasive mechanical ventilation, *MAP* mean atrial pressure, *NLR* neutrophil-to-lymphocyte ratio *PLR* platelet-to-lymphocyte ratio, *PNI* prognostic nutritional index, *SAPSII* simplified acute physiology score II, *SOFA* sequential organ failure assessment, *VIF* variance inflation factor, *WBC* white blood cell

### Construction and internal validation of the prognostic nomogram

**Fig. 2:**
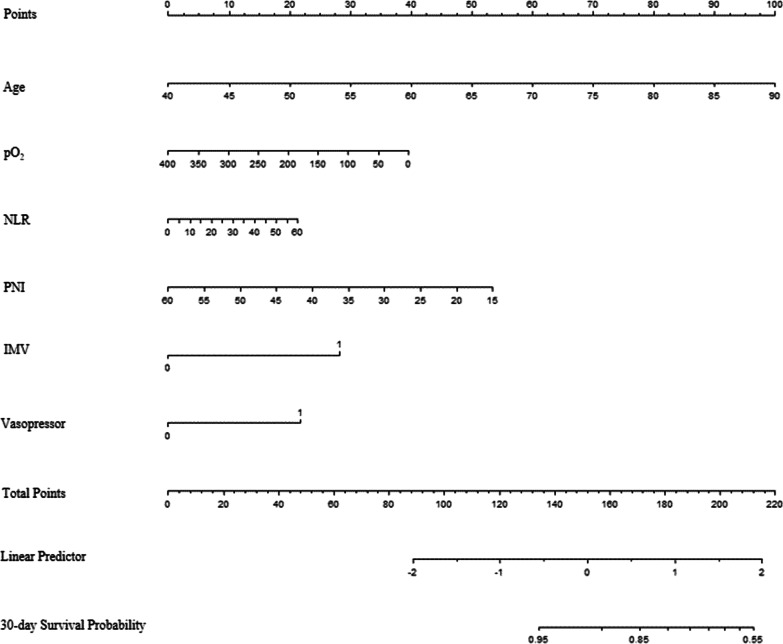
Nomogram to calculate risk score and predict 30-day survival probability in AECOPD patients. Scores were assigned for age, PO_2_, NLR level, PNI level, treatment of IMV and vasopressor by drawing a line upward from the corresponding values to the ‘score’ line. The sum of all these scores, plotted on the ‘Total score’ line, corresponds to predictions of 30-day survival probability. *IMV* invasive mechanical ventilation, *NLR,* neutrophil-to-lymphocyte ratio; *PNI* prognostic nutritional index; *PO*_*2*_ partial pressure of oxygen

**Fig. 3:**
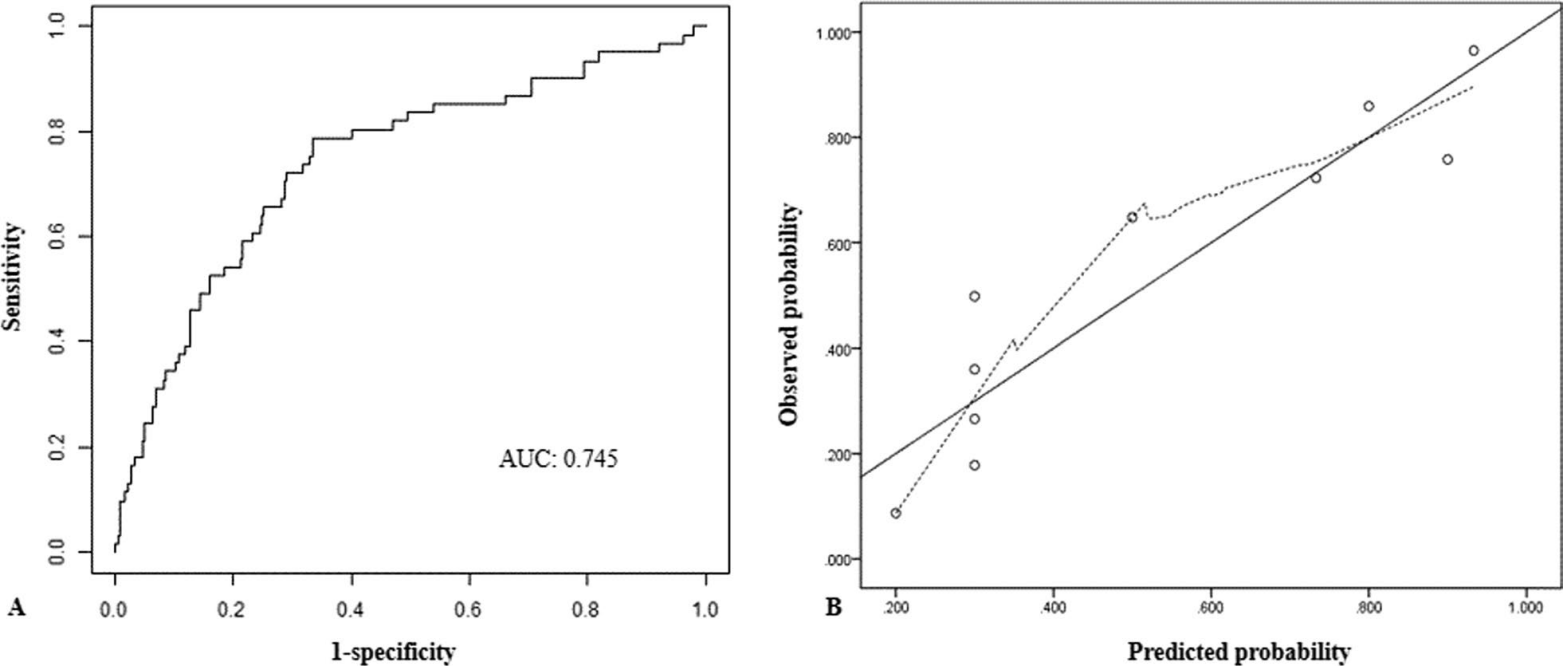
The ROC curve (**A**) and calibration curve (**B**) of the nomogram in predicting 30-day mortality among AECOPD patients in ICU after 1000 bootstrap

**Fig. 4:**
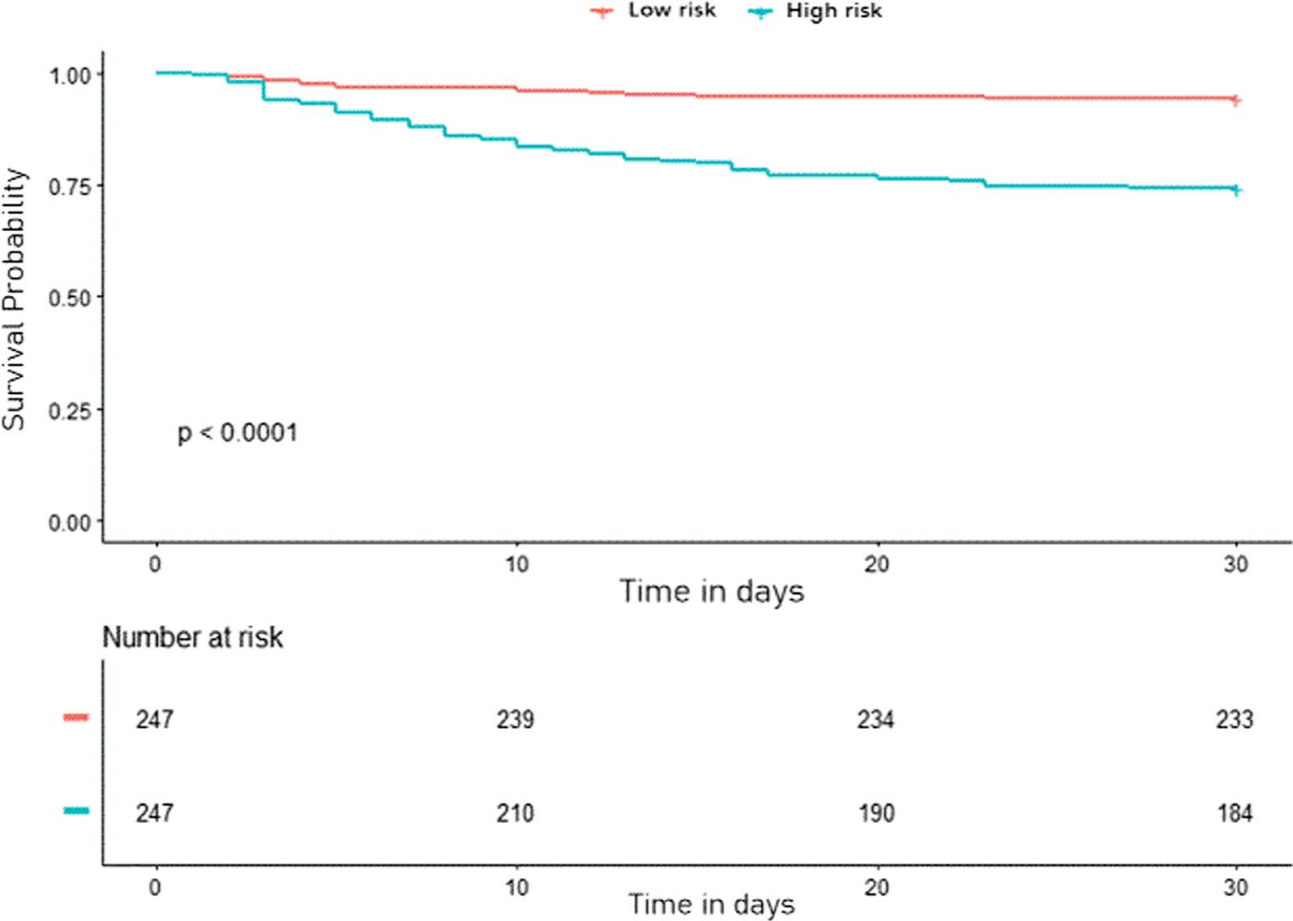
The Kaplan–Meier survival curves classified by low-risk group and high-risk group

The nomogram for predicting the probability of 30-day survival among AECOPD patients was constructed based on the multivariate Cox regression model (Fig. 2). Every specific value of these factors was allocated a score on the points scale. By adding up these scores, the total score was calculated. The discrimination power of the nomogram was evaluated by the C-index values and ROC curves. As the relatively same sample size of our study, we adopted 1000 bootstrap for internal validation. After 1000 samples, the adjusted C-index was 0.745 (95% CI, 0.712 to 0.778) with sensitivity 78.7% and specificity 67.4% (Fig. 3A). The calibration curve showed that the prediction results of the nomogram model were in good agreement with the actual observations (HL test, *P* = 0.147) (Fig. 3B). In addition, the Kaplan–Meier survival curves revealed a significantly lower survival probability in the high-risk group than that in the low-risk group (*P* < 0.001), which indicated the substantial discriminatory power of the nomogram to stratify the risk (Fig. 4).

### Clinical usefulness of the prognostic nomogram

**Fig. 5:**
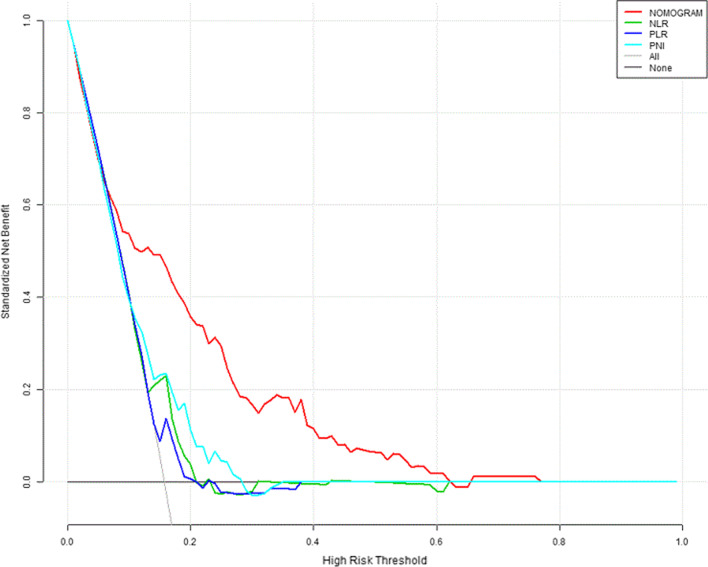
Decision curve analysis for 30-day survival. The x-axis showed the threshold probability. The y-axis represented net benefit. Black line meant that all patients were dead and gray line represented that none patients were dead. The red line displayed the benefit of the nomogram. The green line displayed the benefit of NLR. The blue line displayed the benefit of PLR. The lake blue line displayed the benefit of PNI. *NLR* neutrophil-to-lymphocyte ratio; *PLR* platelet-to-lymphocyte ratio; *PNI* prognostic nutritional index

The DCA curve was plotted to perform a clinical application of this nomogram. Using data from the whole cohort, the DCA showed that if the threshold probability of a patient or doctor is 10%-60%, the prognostic model had a better positive net gain by risk stratification than NLR, PLR and PNI, indicating that it has good potential as a clinical application (Fig. 5).

## Discussion

The prognosis of patients with COPD exacerbations requiring ICU admission is generally poor. In this study, the 30-day mortality was 15.6%, which is consistent with findings of previous studies [4, 5]. Our main purpose was to use clinical data obtained from MIMIC III database to evaluate risk factors associated with 30-day mortality of patients with COPD exacerbation admitted to ICU. Then, we constructed a prognostic nomogram for AECOPD patients using individual patients’ status on admission to ICU. Six risk factors, namely, age, pO_2_, NLR, PNI, IMV and vasopressors use were included to establish a prognostic nomogram. This nomogram demonstrated good discrimination assessed by the C-index and calibration evaluated by HL goodness of fit test. Thus, this nomogram could be efficiently and effectively applied in clinical practice.

The impact of age as a prognostic factor for AECOPD patients is well known. Previous studies have demonstrated the role of age as a determinant of prognosis [4, 15, 16]. Issues related to increasing age, such as frailty, sarcopenia and co-morbidity, might affect prognosis [17]. Besides, elder patients usually present with atypical symptoms, such as muscle weakness, vertigo, confusion and leg edema during severe exacerbations [18]. What’s more, their respiratory system and immune function are impaired and more susceptible to pulmonary infection [6]. Hypoxemia is another frequently recognized prognostic factor. Several studies have reported its impact on poor prognosis in AECOPD patients [19–21]. Additionally, 48.6% of patients treated with IMV in our study and hypoxemia treated with IMV further increase the risk of mortality according to the nomogram. In a study by Brown et al. [22], 38.7% of patients required IMV and multivariate analysis demonstrated that the requirement for IMV was significantly correlated with in-hospital mortality. Cao et al. also found that requirement for IMV was a significant predictor of in-hospital mortality of AECOPD [23]. Vasopressor is the first-line to elevate blood pressure and hypotension occurs more frequently in those non-survivors [24].

The measurement of NLR and PNI are cost-effective and easily performed in clinical laboratories. The neutrophil–lymphocyte ratio, calculated by both neutrophil and lymphocyte counts, is a biomarker to predict systemic inflammation [25]. Several studies have utilized NLR as a marker of inflammation and severity for AECOPD patients. LEE et al. prospectively evaluated the value of NLR in patients with AECOPD, stable disease, and healthy controls. Compared to stable disease and healthy controls, NLR was significantly correlated with AECOPD [26]. Other studies also confirmed that NLR was elevated in AECOPD patients when compared with COPD and healthy controls [27–29]. NLR is also a prognostic biomarker in COPD. Yao et al. enrolled 303 patients with AECOPD. NLR values were significantly higher in non-survivors than those who survived in hospital [28]. Prognostic nutritional index is reflected by serum albumin concentration and peripheral total lymphocyte count. Serum albumin is a hallmark of nutritional status. Hypoalbuminemia could also reflect poor clinical status and persistent inflammation. Several studies have suggested that hypoalbuminemia is associated with increased mortality among AECOPD patients [17, 30, 31]. Lymphocyte count, another biomarker of immune status and inflammation, could predict risk of mortality in AECOPD patients. Lymphocytopenia has been proved to be correlated with increased mortality in COPD patients with acute exacerbation [23, 32]. Then, Peng et al. investigated the role of PNI in predicting mortality among AECOPD patients and they observed that the risk of 30-day mortality significantly increased with the downgraded of PNI [33].

This study still had some limitations. First, the database has large missing data on height and weight, so we could not evaluate the impact of body mass index on mortality. Second, the data for the nomogram were obtained from a single center and selection bias could not be avoided. Third, the generalization of our nomogram should be interpreted with caution due to the absence of external validation. So, we would require multicenter prospective studies to further investigate the clinical practice of our nomogram.

## Conclusion

The nomogram developed in our study could help clinicians to predict risk of 30-day mortality in ICU and assist them to stratify patients and provide appropriate care in clinical setting.

## Data Availability

The datasets used and/or analyzed during the current study are available from the corresponding author on reasonable request. The datasets generated and analyzed during the current study are available in the MIMIC III repository (https://physionet.org/works/MIMICIIIClinicalDatabase/files/).

## Abbreviations

AECOPD: Acute exacerbation of chronic obstructive pulmonary disease
AIC: Akaike information criterion
ALT: Alanine transaminase
AST: Aspartate transaminase
BUN: Blood urea nitrogen
CHD: Coronary heart disease
CKD: Chronic renal disease
DCA: Decision curve analysis
DM: Diabetes mellitus
HR: Hazard ratio
ICU: Intensive care unit
IMV: Invasive mechanical ventilation
IQR: Interquartile range
MAP: Mean atrial pressure
MIMIC: Medical information mart for intensive care
NLR: Neutrophil-to-lymphocyte ratio
pO_2_: Partial pressure of oxygen
pCO_2_: Partial pressure of carbon dioxide
PLR: Platelet-to-lymphocyte ratio
PNI: Prognostic nutritional index
ROC: Receiver operating characteristic
sCr: Serum creatinine
SAPS II: Simplified acute physiology score II
SOFA: Sequential organ failure assessment
VIF: Variance inflation factor
WBC: White blood cell
